# Microglia's Dual Edge: Balancing Neuroprotection and Neurotoxicity in Health, Neurodegeneration, and Neuropsychiatric Disorders

**DOI:** 10.1002/alz70856_096620

**Published:** 2025-12-24

**Authors:** Jitendra Kumar Sinha, Krishna Kumar Singh, Prashant Verma, Madhumita Mahapatra, Shampa Ghosh

**Affiliations:** ^1^ GloNeuro Academy, Noida, Uttar Pradesh, India; ^2^ Symbiosis International (Deemed University), Pune, Maharastra, India; ^3^ BML Munjal University, Gurugram, Haryana, India; ^4^ Delhi Technical Campus, Greater Noida, Uttar Pradesh, India

## Abstract

**Background:**

Microglia, the resident immune cells of the central nervous system (CNS), play a paradoxical role in maintaining neuronal health and contributing to neurodegeneration. Their functions are crucial in the healthy CNS, yet their dysregulation is implicated in various neurodegenerative and neuropsychiatric disorders.

**Method:**

This comprehensive work synthesizes the current understanding of microglia's dual role in neuronal survival, examining both neuroprotective and neurotoxic actions across health, neurodegenerative diseases (e.g., Alzheimer's, Parkinson's), and neuropsychiatric disorders (e.g., depression, schizophrenia).

**Result:**

Key findings highlight the complex mechanisms underlying microglial neuroprotection (including trophic support and regulation of neuroinflammation) and the factors leading to their neurotoxic transformations. We have tried to identify and discuss the therapeutic potential of targeting microglial functions, balancing promise with challenges.

**Conclusion:**

By elucidating microglia's multifaceted roles in neuronal survival, this work informs the development of innovative, microglia‐centric strategies for preventing and treating CNS diseases, ultimately aiming to improve outcomes for affected individuals.

